# Knockdown of UCA1 inhibits viability and glycolysis by suppressing PKM2 expression through the mTOR pathway in non-small cell lung cancer cells

**DOI:** 10.1039/c8ra00860d

**Published:** 2018-03-19

**Authors:** Xuguang Wang, Xian-En Fa

**Affiliations:** Department of Thoracic Surgery, The First Affiliated Hospital of Zhengzhou University Zhengzhou 450052 P. R. China; Department of Cardiothoracic Surgery, The Second Affiliated Hospital of Zhengzhou University No. 2 Jingba Road Zhengzhou 450014 P. R. China xianenfazzu@163.com

## Abstract

LncRNA urothelial carcinoma associated 1 (UCA1) was reported to be upregulated in non-small cell lung cancer (NSCLC) tissues and contributed to NSCLC progression. Additionally, it has been proposed that the oncogenic role of UCA1 may be related to glucose metabolism in bladder cancer. However, whether and how UCA1 regulates glucose metabolism in the progression of NSCLC remains unknown. Our results showed that knockdown of UCA1 inhibited the viability of NSCLC cells. UCA1 silencing suppressed glycolysis of NSCLC cells by reducing the glucose consumption and lactate production. Additionally, knockdown of UCA1 suppressed PKM2 expression and the mTOR pathway in NSCLC cells. Mechanistically, PKM2 knockdown suppressed the effects of UCA1 on viability and glycolysis of NSCLC cells and inhibition of the mTOR pathway suppressed the effects of UCA1 on viability, glycolysis, and PKM2 expression in NSCLC cells. In conclusion, knockdown of UCA1 inhibited viability and glycolysis by suppressing PKM2 expression maybe through the mTOR pathway in NSCLC cells, providing a novel insight into the molecular mechanism of UCA1 involved in the regulation of glucose metabolism in NSCLC cells.

## Abstract 2

LncRNA urothelial carcinoma associated 1 (UCA1) was reported to be upregulated in non-small cell lung cancer (NSCLC) tissues and contributed to NSCLC progression.

## Graphical abstract

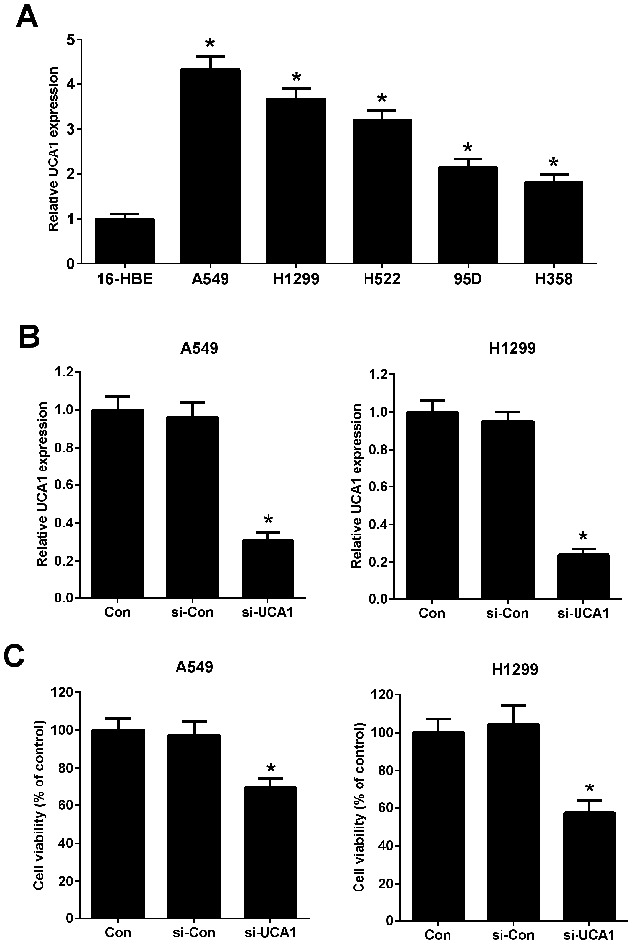

## Introduction

Lung cancer is one of the most frequently diagnosed malignancies and the leading cause of cancer-associated mortality and morbidity worldwide, accounting for more than 1.4 million deaths annually.^1^ Non-small cell lung cancer (NSCLC) represents the most predominant pathological type of lung cancer, comprising up to 85% of all lung cancer cases.^2^ Although the incidence rate is continuously decreasing with the improvement of early diagnosis and clinical treatments, the five-year survival rate of patients with NSCLC is still approximately 15% due to disease recurrence or metastasis.^3^ It has been suggested that the molecular basis of NSCLC is complicated and heterogeneous process, which is involved in dysregulation of multiple oncogenes and tumor-suppressive genes.^4,5^ Thus, investigation of the molecular mechanisms underlying NSCLC development and progression is necessary to improve the diagnosis and treatment of NSCLC.

Long non-coding RNAs (lncRNAs) are commonly defined as a highly heterogeneous group of transcripts longer than 200 nucleotides in length and regulate gene expression at transcriptional, posttranscriptional, and epigenetic levels.^6^ More recently, substantive studies showed that lncRNAs have the potential function in a wide range of pathological and physiological processes, including cellular differentiation, proliferation, glucose metabolism, and tumorigenesis.^7,8^ Convincing evidence has demonstrated that a large amount of lncRNAs are frequently dysregulated in human cancers and contribute to tumor development and progression.^9^ Currently, multiple lncRNAs, such as lncRNA HNF1A-AS1,^10^ MIAT,^11^ and MALAT1,^12^ have been identified to be involved in NSCLC progression. Human urothelial carcinoma associated 1 (UCA1), located at chromosome 19p13.12 with 1.4 kb in length, is a well-characterized lncRNA that was initially identified in human bladder carcinoma.^13^ UCA1 has been reported to be aberrantly overexpressed and plays an oncogenic role in various cancers, such as hepatocellular carcinoma,^14^ bladder cancer,^15^ and colorectal cancer.^16^ Previous studies also revealed that UCA1 was highly expressed in NSCLC tissues and served as a potential biomarker for diagnosis of NSCLC and upregulated UCA1 contributed to NSCLC progression by functioning as an oncogene.^17,18^ It has been proposed that the oncogenic role of UCA1 may be related to glucose metabolism in bladder cancer.^19,20^ However, whether and how UCA1 regulates glucose metabolism in the progression of NSCLC remain to be further explored.

Pyruvate kinase isoenzyme M2 (PKM2) is a key rate-limiting glycolytic enzyme which catalyzes the conversion of phosphoenolpyruvate (PEP) and adenosine diphosphate (ADP) to pyruvate and adenosine triphosphate (ATP) in the last step of glycolysis.^21^ Increasing evidence has shown that PKM2 acts as a key regulator of glucose metabolism and tumor growth, invasion and metastasis.^22^ Cancer cells expressing PKM2 grow more rapidly compared to cells expressing PKM1, indicating that PKM2, but not PKM1, promotes the glycolysis and tumor growth.^23^ Notably, it was reported that PKM2 knockdown inhibited glycolysis and lipid synthesis, thereby suppressing cell proliferation and invasion in lung cancer cells.^24^

In the present study, we aimed to investigate whether UCA1 exerted its oncogenic role and regulated glucose metabolism in NSCLC *via* PKM2 and the underlying mechanism.

## Materials and methods

### Cell culture and transfection

The normal bronchial epithelial cell line 16-HBE and human lung cancer cell lines (A549, H1299, H522, 95D, and H358) were purchased from the American Type Culture Collection (ATCC, Manassas, VA, USA). Cells were cultivated in Dulbecco's Modified Eagle's Medium (DMEM; Thermo Fisher Scientific, Waltham, MA, USA) supplemented with 10% fetal bovine serum (FBS, HyClone Laboratories, Logan, UT, USA), 100 U ml^−1^ penicillin, and 100 μg ml^−1^ streptomycin (Gibco, Grand Island, NY, USA) in a 37 °C humidified incubator containing 5% CO_2_. To attenuate UCA1 or PKM2 expression, siRNAs specifically targeting UCA1 (si-UCA1) or PKM2 (si-PKM2) were synthesized by Ribobio (Guangzhou, China), with nonspecific oligonucleotides (si-Con) as a control. To overexpress UCA1, the full length of UCA1 sequence was synthesized and cloned into pcDNA3.1 vector (GenePharma, Shanghai, China) to generate pcDNA-UCA1, with pcDNA3.1 empty vector (pcDNA-NC) as a control. A549 and H1299 cells at 80% confluence were transfected with siRNAs or pcDNAs using Lipofectamine™ 2000 reagent (Invitrogen, Carlsbad, CA, USA). Cells were collected for subsequent analyses at 48 h posttransfection.

### Quantitative reverse transcription polymerase chain reaction (qRT-PCR)

The total RNA was extracted from A549 or H1299 cells using TRIzol reagent (Invitrogen). Total RNA samples were reversely transcribed into first-strand cDNA using M-MLV reverse transcriptase (Promega, Madison, WI, USA). The expression of UCA1 was examined using the SYBR-Green PCR Master Mix Kit (Takala, Dalian, China) on an ABI 7500 thermocycler (Thermo Fisher Scientific), with GAPDH as an endogenous control. The relative fold change of gene expression was calculated using the 2^−ΔΔ*C*_t_^ method. UCA1 primers were: forward, 5′-ACGCTAACTGGCACCTTGTT-3′, reverse, 5′-TGGGGATTACTGGGGTAGGG-3′. PKM2 primers were: forward, 5′-CAGAGGCTGCCATCTACCAC-3′, reverse, 5′-CCAGACTTGGTGAGGACGAT-3′. GAPDH primers were: forward, 5′-ACAACTTTGGTATCGTGGAAGG-3′ and reverse, 5′-GCCATCACGCCACAGTTTC-3′.

### Cell viability assay

Cell viability was assessed by 3-(4,5-dimethylthiazol-2-yl)-2,5-diphenyltetrazolium bromide (MTT) assay. Briefly, 48 h after transfection, A549 and H1299 cells were seeded in a 96-well plate at a density of 5000 cells per well, 10 μl MTT solution (5 mg ml^−1^; Sigma, St. Louis, MO, USA) was added to each well and incubated for another 4 h in the dark. Then, the supernatant was discarded and 100 μl dimethyl sulfoxide (DMSO) was added to each well to resolve formazan crystals. The optical density at 490 nm was measured by a microplate reader (Molecular Devices, Sunnyvale, CA, USA). After transfection with pcDNA-NC or pcDNA-UCA1 for 24 h, A549 and H1299 cells were treated with 2.5 mM 2-deoxy-d-glucose (2-DG) or rapamycin (25 or 50 nM) for another 24 h. Cell viability was evaluated as described above.

### Measurement of lactate production and glucose consumption

After treatment, the supernatant of culture medium was harvested for measurement of glucose and lactate concentration. The glucose and lactate levels in the supernatant were determined using a Glucose Uptake Colorimetric Assay Kit (Sigma) and the Lactate Assay Kit (Biovision, Mountain View, CA, USA), respectively. The results were normalized to control.

### Western blot

The treated A549 or H1299 cells were collected, washed with PBS and lysed in RIPA buffer (Beyotime, Shanghai, China). The protein concentration was quantified using a Protein BCA Assay Kit (Bio-Rad, Hercules, California, USA). Equal amount of protein samples (30 μg) was fractionated by 10% sodium dodecyl sulfate polyacrylamide gel electrophoresis (SDS-PAGE) and then transferred to a polyvinylidene fluoride (PVDF) membrane (EMD Millipore, Billerica, MA, USA). After blocking with 5% skim milk in 0.05% TBS-Tween-20 (v/v) for 1 h, the membranes were incubated overnight at 4 °C with the primary antibodies against PKM2 (Sigma), phosphorylated mammalian target of rapamycin (p-mTOR) (Abcam, Cambridge, UK), mTOR (Abcam), or β-actin (Cell Signaling Technology, Danvers, MA, USA), followed by incubation with horseradish peroxidase (HRP)-linked secondary antibody (Abcam) for 2 h. The signal bands were visualized by an electrochemiluminescence kit (Pierce Biotechnology, Rockford, IL, USA).

### Statistical analysis

Data are shown as mean ± standard deviation (SD) (*n* = 3). The results were analyzed using SPSS 20.0 software (SPSS, Chicago, IL, USA) with one-way analysis of variance. *P* < 0.05 was considered as statistically significant.

## Results

### Knockdown of UCA1 inhibited viability of NSCLC cells

**Fig. 1:**
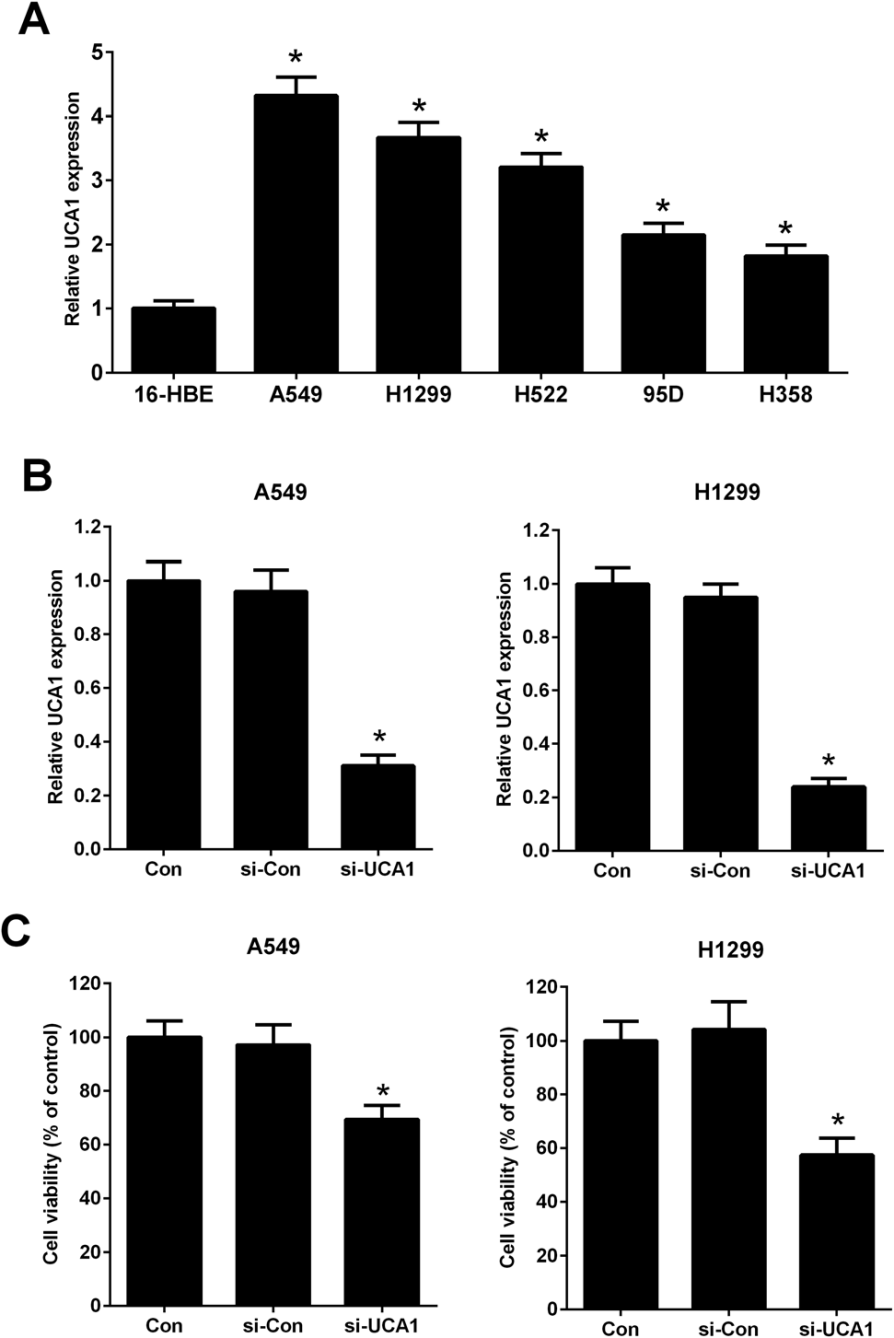
The effect of UCA1 on the viability of NSCLC cells. (A) qRT-PCR was conducted to determine the expression of UCA1 in a normal human lung bronchial epithelial cell line (16-HBE) and human lung cancer cell lines (A549, H1299, H522, 95D, and H358). *P < 0.05 *vs.* 16-HBE. (B) UCA1 expression in transfected A549 and H1299 cells was determined by qRT-PCR analysis. A549 and H1299 cells were transfected with si-UCA1 or si-Con and incubated for 48 h. (C) MTT assay was conducted to evaluate viability of A549 and H1299 cells 48 h after transfection. *P < 0.05 *vs.* control.

It has been previously demonstrated that UCA1 was upregulated in NSCLC cells. The expression of UCA1 in a normal human lung bronchial epithelial cell line (16-HBE) and human lung cancer cell lines (A549, H1299, H522, 95D, and H358) was determined using qRT-PCR. The results showed that the expression levels of UCA1 in A549, H1299, H522, 95D, and H358 cells were increased compared to those in 16-HBE cells (Fig. 1A). A549 and H1299 cells were used for the subsequent experiments due to higher expression levels of UCA1. We performed loss-of-function approaches to confirm the functional effect of UCA1 on the viability of NSCLC cells. qRT-PCR results showed that, after transfection with si-UCA1, UCA1 expression was reduced in A549 and H1299 cells when compared with si-Con group (Fig. 1B). MTT assay manifested that cell viability was blocked following UCA1 knockdown in A549 and H1299 cells compared with si-Con group (Fig. 1C), suggesting that UCA1 knockdown impeded NSCLC cell viability.

### Knockdown of UCA1 suppressed glycolysis of NSCLC cells

**Fig. 2:**
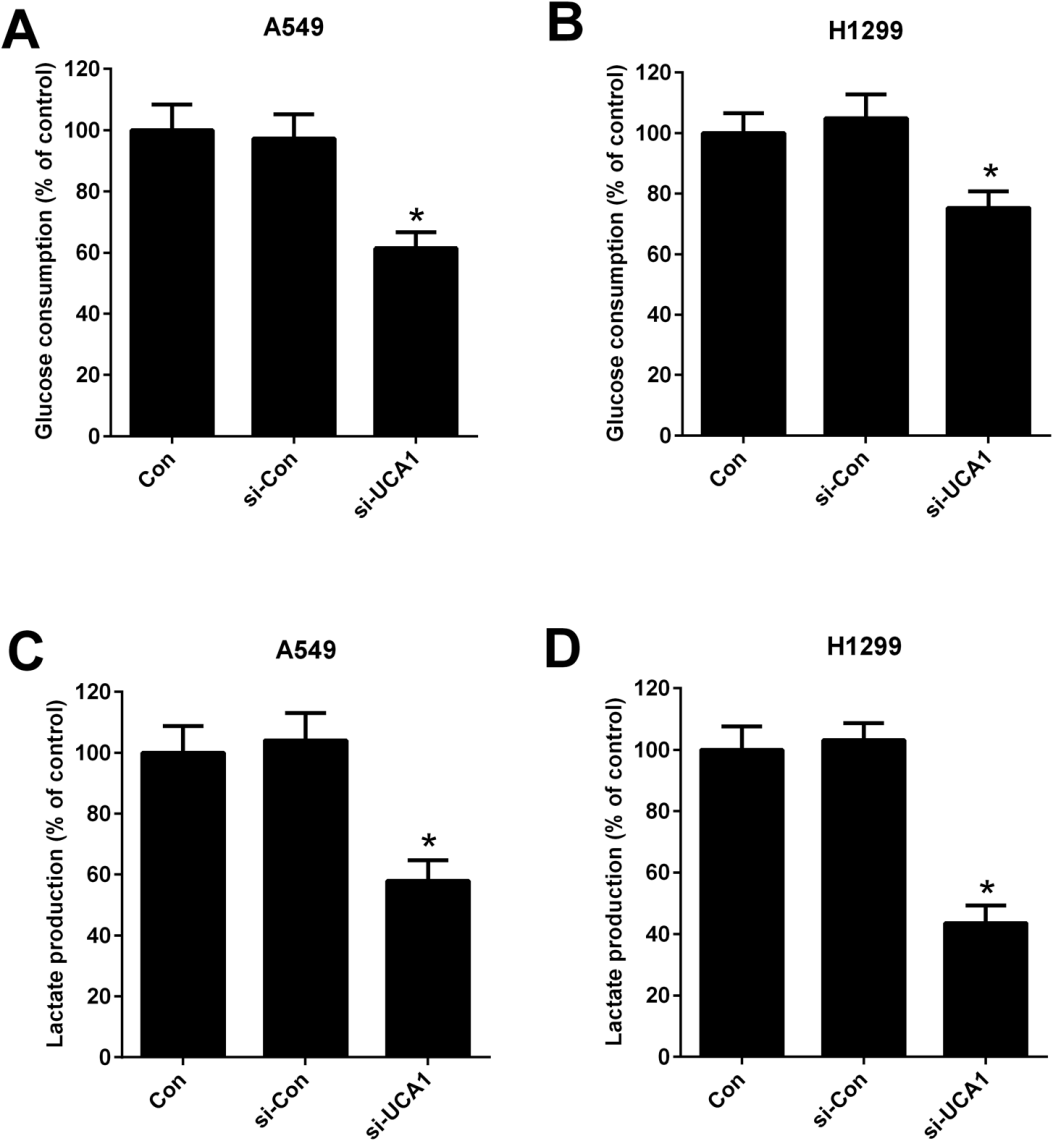
The effect of UCA1 on glycolysis in NSCLC cells. (A and B) The glucose consumption in A549 and H1299 cells transfected with si-UCA1 or si-Con. (C and D) Lactate production in A549 and H1299 cells transfected with si-UCA1 or si-Con for 48 h. *P < 0.05 *vs.* control group.

Considering the oncogenic role of UCA1 and that the alteration metabolism is associated with the abnormal proliferation of cancer cells,^25^ we analyzed the effect of UCA1 on glycolysis in NSCLC cells by measuring the glucose consumption and lactate production. As shown in Fig. 2A–D, UCA1 depletion triggered a decline of glucose consumption and lactate production in A549 and H1299 cells *versus* si-Con group, suggesting an inhibition of glycolysis by UCA1 silencing in NSCLC cells.

### UCA1 regulated viability of NSCLC cells by modulation of glycolysis

**Fig. 3:**
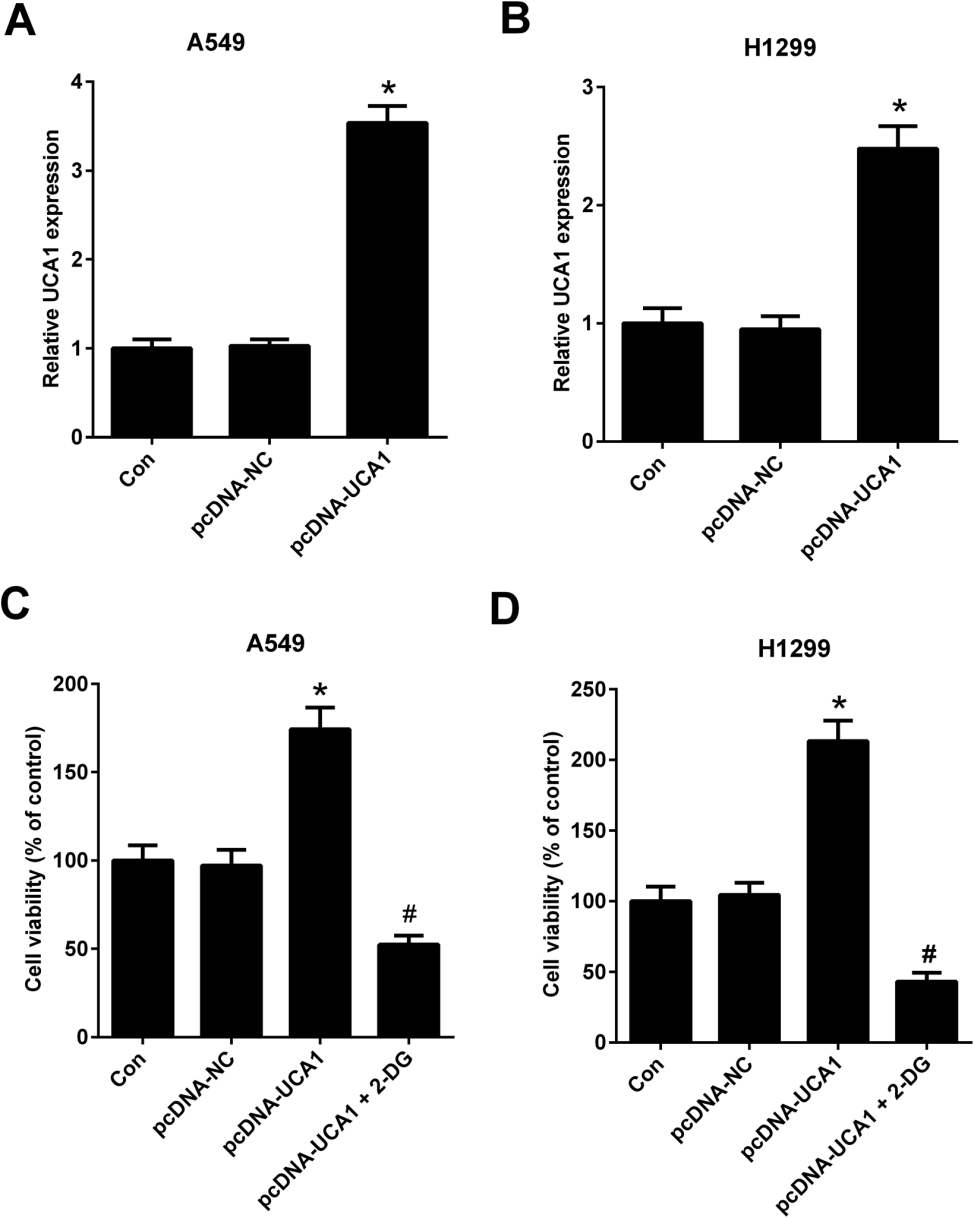
Forced expression of UCA1 promoted viability of NSCLC cells by enhancing glycolysis. UCA1 expression in A549 (A) and H1299 (B) cells transfected with pcDNA-NC or pcDNA-UCA1 for 48 h was determined by qRT-PCR analysis. *P < 0.05 *vs.* Con. (C and D) MTT assay was used to assess cell viability in A549 and H1299 cells after transfection with pcDNA-NC or pcDNA-UCA1 for 24 h, followed by treatment with 2DG for another 24 h. *P < 0.05 *vs.* pcDNA-NC. ^#^*P* < 0.05 *vs.* pcDNA-UCA1.

To further investigate the effect of altered glycolysis by dysregulated UCA1 on the viability of NSCLC cells, A549 and H1299 cells transfected with pcDNA-NC or pcDNA-UCA1 were treated with 2-DG, a glycolytic inhibitor, for 24 h. Firstly, qRT-PCR results proved that UCA1 expression was elevated in A549 (A) and H1299 (B) cells transfected with pcDNA-UCA1. MTT assay suggested that ectopic overexpression of UCA1 led to an enhancement of viability of A549 and H1299 cells, while this effect was attenuated by the addition of 2-DG (Fig. 3C and D). Therefore, we concluded that forced expression of UCA1 promoted viability of NSCLC cells by enhancing glycolysis.

### Knockdown of UCA1 suppressed PKM2 expression in NSCLC cells

**Fig. 4:**
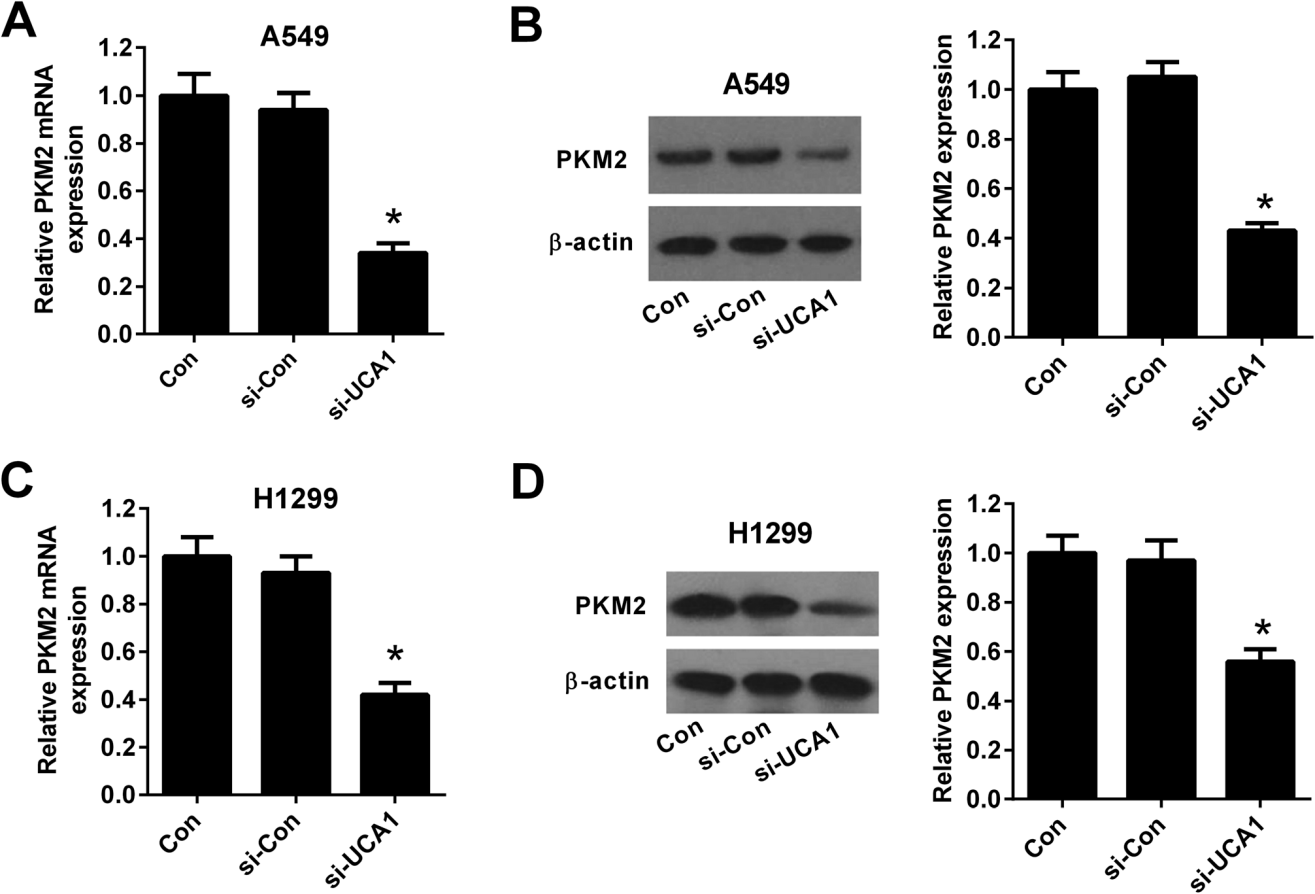
The effect of UCA1 knockdown on PKM2 expression in NSCLC cells. qRT-PCR and western blot were conducted to determine the mRNA and protein levels of PKM2 in A549 (A and B) and H1299 (C and D) cells transfected with si-UCA1 or si-Con for 48 h. *P < 0.05 *vs.* si-Con.

To explore the molecular mechanism by which UCA1 regulated the viability of NSCLC cells *via* modulation of glycolysis, we detected the expression of PKM by qRT-PCR and western blot. The results demonstrated that the mRNA and protein levels of PKM2 was suppressed after UCA1 was silenced by si-UCA in both A549 (Fig. 4A and B) and H1299 cells (Fig. 4C and D).

### Knockdown of UCA1 inhibited the mTOR pathway in NSCLC cells

**Fig. 5:**
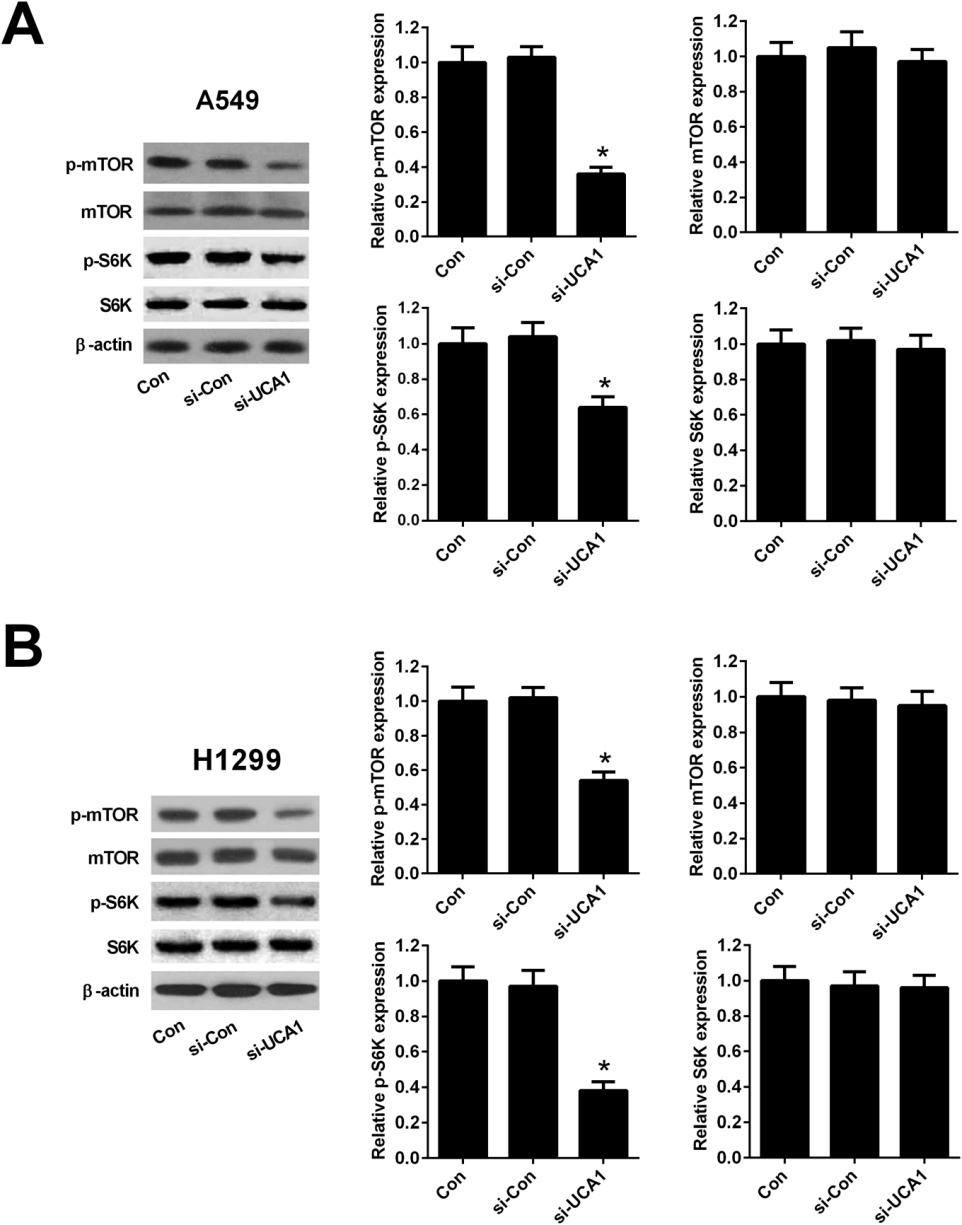
Knockdown of UCA1 inhibited the mTOR pathway in NSCLC cells. Western blot was performed to detect the protein levels of p-mTOR, mTOR, p-S6K, and S6K in A549 (A) and H1299 (B) cells transfected with si-UCA1 or si-Con. *P < 0.05 *vs.* si-Con.

Since previous studies demonstrated that PKM2 could interact with mTOR signaling, which is a master regulator of cell metabolism,^26^ we further detected the effect of UCA1 on the mTOR signaling in NSCLC cells. As shown in Fig. 5A and B, western blot analysis implicated that the protein levels of p-mTOR reduced 64% in A549 and 46% in H1299 cells after transfection with si-UCA1 for 48 h. The protein levels of p-S6K reduced 36% in A549 and 62% in H1299 cells after transfection with si-UCA1 for 48 h, but UCA1 knockdown had no influence on mTOR and S6K expression. These data suggested that knockdown of UCA1 inhibited the mTOR pathway in NSCLC cells.

### PKM2 knockdown suppressed the effects of UCA1 on viability and glycolysis of NSCLC cells

**Fig. 6:**
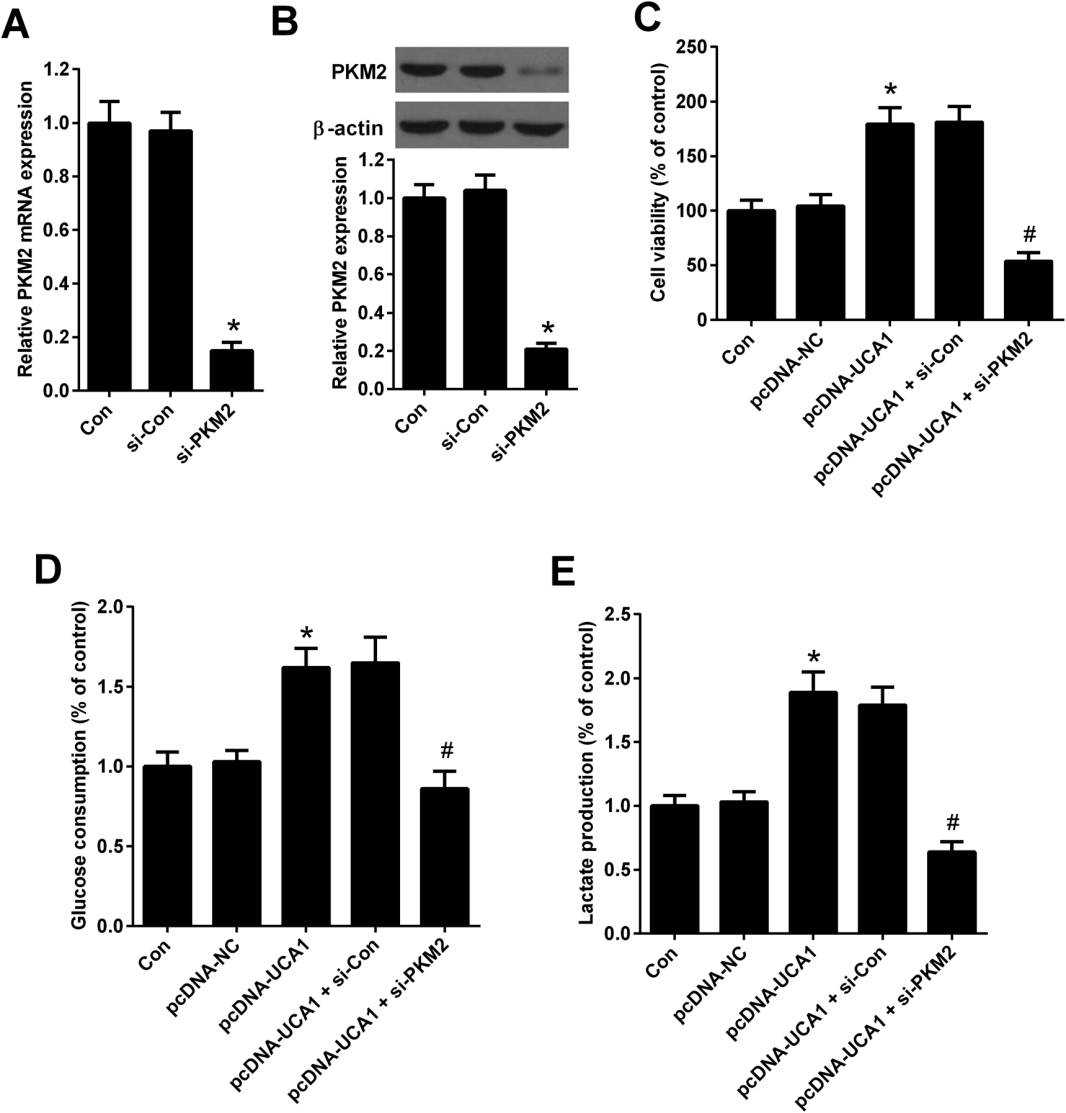
PKM2 knockdown attenuated the effects of UCA1 on viability and glycolysis of NSCLC cells. (A) PKM2 mRNA expression in A549 cells 48 h after transfection with si-PKM2 or si-Con was determined by qRT-PCR. *P < 0.05 *vs.* si-Con. (B) PKM2 expression in A549 cells 48 h after transfection with si-PKM2 or si-Con was determined by western blot. *P < 0.05 *vs.* si-Con. (C) MTT assay was performed to detect the viability of A549 cells 48 h after transfection with pcDNA-UCA1, pcDNA-NC, or combined with si-PKM2 or si-Con. *P < 0.05 *vs.* pcDNA-NC. ^#^*P* < 0.05 *vs.* pcDNA-UCA1 + si-Con. (D and E) Glucose consumption and lactate production were measured in A549 cells 48 h after transfection with pcDNA-UCA1, pcDNA-NC, or combined with si-PKM2 or si-Con. *P < 0.05 *vs.* pcDNA-NC. ^#^*P* < 0.05 *vs.* pcDNA-UCA1 + si-Con.

To explore whether the effects of PKM2 on the increased viability and glycolysis of NSCLC cells induced by UCA1, rescue experiments were performed in A549 cells by transfection of with pcDNA-UCA1, pcDNA-NC, or combined with si-PKM2 or si-Con. qRT-PCR and western blot analysis first confirmed the high knockdown efficiency of si-PKM2 in A549 cells (Fig. 6A and B). MTT assay demonstrated that pcDNA-UCA1 transfection facilitated cell viability in A549 cells relative to pcDNA-NC group, but was counteracted after cotransfection with pcDNA-UCA1 and si-PKM2 (Fig. 6C). As shown in Fig. 6D and E, increased UCA1 expression enhanced glycolysis levels in A549 cells, as evidenced by the increased glucose consumption and lactate production, while this effect was weakened by PKM2 knockdown. These results demonstrated that PKM2 knockdown attenuated the effects of UCA1 on viability and glycolysis of NSCLC cells.

### Inhibition of mTOR pathway suppressed the effects of UCA1 on viability, glycolysis, and PKM2 expression in NSCLC cells

**Fig. 7:**
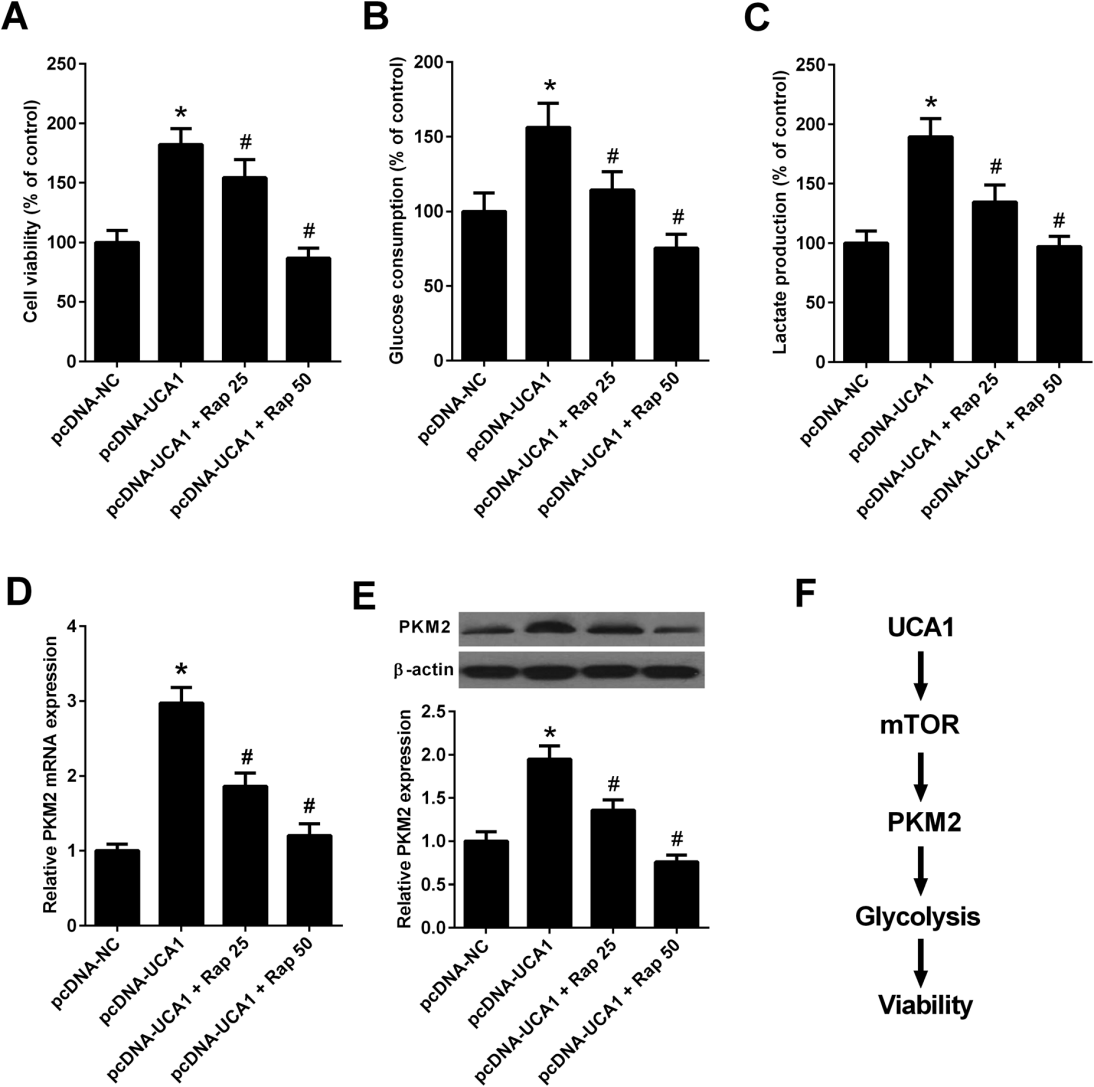
UCA1 regulated viability, glycolysis, and PKM2 expression through the mTOR pathway in NSCLC cells. A549 cells were transfected with pcDNA-NC or pcDNA-UCA1 for 24 h, followed by exposure to 25 nM or 50 nM rapamycin for another 24 h. (A) Cell viability of A549 cells was assessed by MTT assay. (B and C) Glucose consumption and lactate production in A549 cells were detected. (D) The mRNA levels of PKM2 in A549 cells were determined by qRT-PCR. (E) The protein levels of PKM2 in A549 cells were examined by western blot. (F) Schematic diagram summarizing how UCA1 regulates viability, glycolysis, and PKM2 expression in NSCLC cells. Rap, rapamycin. *P < 0.05 *vs.* pcDNA-NC. ^#^*P* < 0.05 *vs.* pcDNA-UCA1.

Since the above results revealed that UCA1 knockdown inhibited the mTOR pathway in NSCLC cells, we then analyzed whether the mTOR pathway could affect the effects of UCA1 on viability, glycolysis, and PKM2 expression. A549 cells were transfected with pcDNA-NC or pcDNA-UCA1, followed by exposure to 25 nM or 50 nM rapamycin, an inhibitor of the mTOR pathway. MTT assay implied that administration with 25 nM or 50 nM rapamycin reversed overexpression of UCA1-mediated promotion of cell viability in A549 cells (Fig. 7A). Inhibition of the mTOR pathway by 25 nM or 50 nM rapamycin attenuated the enhanced glucose consumption and lactate production induced by exogenous UCA1 in A549 cells (Fig. 7B and C). Furthermore, qRT-PCR and western blot analyses manifested that ectopic expression of UCA1 increased the mRNA and protein levels of PKM2 in A549 cells, which was attenuated following the addition of 25 nM or 50 nM rapamycin (Fig. 7D and E). Collectively, these findings suggested that UCA1 regulated viability, glycolysis, and PKM2 expression through the mTOR pathway in NSCLC cells.

## Discussion

In recent years, an increasing amount of literatures have indicated that the high expression of UCA1 contributes to development of a variety of tumors, including NSCLC.^14,18,27^ A previous study showed that knockdown of UCA1 enhanced tamoxifen sensitivity of tamoxifen-resistant breast cancer cells and induced more apoptotic cells through attenuating the activation of Wnt/β-catenin pathway.^28^ Another study demonstrated that knockdown of UCA1 inhibited glioma cell viability, migration, and invasion *via* reduction of epithelial–mesenchymal transition and inactivation of the Akt pathway.^29^ More interestingly, UCA1 has been documented to play a positive role in cancer cell glucose metabolism by upregulating hexokinase 2 (HK2) through the cascade of mTOR-STAT3/microRNA-143.^19^ However, whether and how UCA1 regulated NSCLC cell glucose metabolism remain largely elusive. In our study, we provided the first evidence that UCA1 knockdown impeded the viability and glycolysis of NSCLC cells by suppressing PKM2 expression through the mTOR pathway in NSCLC, shedding light on the specific regulatory mechanism of UCA1 in NSCLC.

The Warburg effect, characterized by increased glucose consumption and lactate production even in sufficient oxygen state,^30^ is considered as a well-recognized hallmark of cancer cells.^31^ Accumulating evidence indicates that reprogrammed energy metabolism, including hyperactive aerobic glycolysis, is frequently observed in human cancers, which is accompanied by the facilitated cell proliferation and growth.^32^ Several studies have suggested the involvement of lncRNAs in cancer metabolic reprogramming. For instance, lncRNA PTV1 contributed to osteosarcoma cell glucose metabolism, cell proliferation and motility through the miR-497/HK2 pathway.^33^ LncRNA IGFBP4 promoted lung cancer cell proliferation and metastasis through possible mechanism of reprogramming tumor cell energy metabolism.^34^ Moreover, lncRNA CASC8 suppressed bladder cancer cell proliferation *via* regulating glycolysis.^35^ In our study, we demonstrated that UCA1 knockdown significantly inhibited and forced expression of UCA1 promoted NSCLC cell viability. Additionally, UCA1 knockdown suppressed glycolysis in NSCLC cells, as evidenced by the reduced glucose consumption and lactate production. Importantly, we found that exogenous UCA1 promoted cell viability by accelerating glycolysis, suggesting a link between UCA1 and altered glycolysis in the development of NSCLC cells.

Alteration of metabolism in cancer tissues and cells is partially attributed to the upregulation of PKM2.^36^ As a critical driver of aerobic glycolysis in cancer cells, PKM2 results in enhanced glucose consumption and lactate production in order to meet the energy demands of tumor growth.^22^ PKM2 has been documented to be frequently overexpressed in many types of tumors, including breast, lung, and colon cancers, and contribute to the malignant phenotypes of cancers.^22,37^ Accordingly, PKM2 is evaluated as a tumor diagnostic biomarker and particularly as a potential therapeutic target for cancer treatment.^38^ siRNA-mediated knockdown of PKM2 inhibited cell proliferation, induced apoptosis, caused cell cycle arrest at the G0/G1 phase, and suppressed cell migration and invasion in ovarian cancer cells.^39^ siRNA-mediated PKM2 knockdown also significantly suppressed the proliferation, glucose uptake, ATP generation, fatty acid synthesis, and the expression of the glucose transporter GLUT1, ATP citrate lyase, matrix metalloproteinase 2 (MMP2), and vascular endothelial growth factor (VEGF) in A549 cells, while the mitochondrial respiratory capacity of the cells increased, indicating that PKM2 knockdown inhibited cell proliferation and invasion by suppressing glycolysis and lipid synthesis.^23^ Moreover, enhanced PKM2 expression contributed to the carcinogenesis and development of gastric cancer in part by regulating cancer-specific metabolism.^40^ In our study, we suggested that UCA1 silencing inhibited PKM2 expression in NSCLC cells. Rescue experiments demonstrated that PKM2 knockdown suppressed the effects of UCA1 on viability and glycolysis of NSCLC cells, suggesting that UCA1 knockdown impeded the viability and glycolysis of NSCLC cells by inhibiting PKM2 expression.

It is well-known that the phosphatidylinositol-3-kinase (PI3K)/protein kinase B (Akt)/mTOR signaling pathway is an essential pathway for multiple cellular processes, including cell growth, survival, and several metabolic processes including glycolysis.^41^ Activation of mTOR could enhance the expressions of glycolytic enzymes including PKM2, and disruption of PKM2 suppresses oncogenic mTOR-mediated tumorigenesis.^42^ A previous study demonstrated that inactivation of mTOR by rapamycin attenuated the effect of UCA1 on glycolysis in bladder cancer cells.^19^ In our study, we suggested that UCA1 knockdown inhibited the mTOR pathway in NSCLC cells. Mechanistically, we further revealed that inhibition of mTOR pathway by rapamycin suppressed the effects of UCA1 on viability, glycolysis, and PKM2 expression in NSCLC cells. Therefore, we concluded that knockdown of UCA1 inhibited the glycolysis of NSCLC cells by suppressing PKM2 expression through inactivation of the mTOR pathway, ultimately facilitating cell viability (Fig. 7F).

## Conclusion

In conclusion, our study demonstrated for the first time that UCA1 knockdown hindered the viability and glycolysis of NSCLC cells by inhibiting PKM2 expression maybe through inactivation the mTOR pathway, providing a novel insight into the molecular mechanism of UCA1 involved in the regulation of glucose metabolism.

## Conflicts of interest

The authors declare no conflict of interest.

## Supplementary Material
